# Cystatin C-Based Renal Function in Predicting the Long-Term Outcomes of Chronic Total Occlusion After Percutaneous Coronary Intervention

**DOI:** 10.3389/fcvm.2020.586181

**Published:** 2020-11-09

**Authors:** Bolin Li, Jie Rong, Bobo Wang, Ke Gao, Xing Wen, Hongbing Li, Lele Cheng, Yi-Ming Hua, Shanshan Li, Zhijie Jian, Yujing Zhang, Hui Huang, Youlong Pan, Yue Wu, Xiao-Zhen Zhuo

**Affiliations:** ^1^Department of Cardiology, The First Affiliated Hospital of Xi'an Jiaotong University, Xi'an, China; ^2^Department of Encephalopathy, Affiliated Hospital of Shaanxi University of Chinese Medicine, Xianyang, China; ^3^Health Science Center, School of Pharmacy, Xi'an Jiaotong University, Xi'an, China; ^4^The Center of Gastrointestinal and Minimally Invasive Surgery, The Third People's Hospital of Chengdu, Chengdu, China; ^5^Cardiovascular Department, The General Hospital of Ningxia Medical University, Yinchuan, China; ^6^Cardiovascular and Cerebrovascular Diseases Hospital of Qinghai Province, Xining, China

**Keywords:** cystatin C, creatinine, estimated glomerular filtration rate, chronic total occlusion, all-cause mortality, cardiac death

## Abstract

Renal function estimated by various biomarkers predicting for adverse cardiovascular events has not been well-identified in received percutaneous coronary intervention (PCI) for chronic total occlusion (CTO), the advanced stages of atherosclerosis. We aim to determine whether the serum cystatin C-based-estimated glomerular filtration rate (eGFR) can have an improved predictive value in patients with CTO lesions undergoing PCI as compared with multiple creatinine-based estimates of kidney function. Six hundred and seventy-one patients received CTO PCI were retrospectively included in the study. The eGFR was calculated by modification of diet in renal disease equation for Chinese (cMDRD) and Chronic Kidney Disease Epidemiology Collaboration (CKD-EPI) equations at baseline, respectively. Then, the cohort was categorized into three groups according to standard KDIGO kidney stages based on eGFR. The primary endpoint was all-cause mortality, and the secondary endpoint was cardiac death. Strikingly, cystatin C-based eGFR showed a better performance with the greater area being under the receiver operating characteristic (ROC) curve (0.73 for all-cause mortality and 0.73 for cardiac death, separately) and a better assessment for survival free from adverse event across renal levels among four eGFR equations. Compared with eGFR calculated by other formulas, serum cystatin C-based eGFR showed the highest prognostic value for both all-cause mortality (adjusted HR 3.6, 95% CI 1.6–8.1, *P* = 0.002) and cardiac death (adjusted HR 2.9, 95% CI 1.0–8.1, *P* = 0.028). Moreover, cystatin C-based eGFR significantly improved the risk reclassification of event with a high value of net reclassification improvement and integrated discrimination improvement. This study may prove that cystatin C-based eGFR is a better predictor of both all-cause mortality and cardiac death than other equations in populations with CTO undergoing PCI.

## Introduction

Renal insufficiency has been found to increase the incidence of both cardiovascular diseases and adverse outcome (1). Estimated glomerular filtration rate (eGFR) has been widely used in clinical practice. Current recommendations and guidelines (2) have pointed out that the Chronic Kidney Disease Epidemiology Collaboration (CKD-EPI) creatinine equation can be applied to estimate GFR with better accuracy than the modification of Diet in Renal Disease (MDRD) equation, as the actual GFR in patients with preserved renal function may be underestimated by using MDRD (3, 4).

Recently, a number of endogenous biomarkers, especially cystatin C, have been established and used to estimate GFR. Cystatin C is a 13-kDa protein and a member of the family of competitive lysosomal cysteine protease inhibitors. It can be freely filtered by the glomerulus. Compared to creatinine-based eGFR, cystatin C depends less on health status, muscle mass, or other demographic characteristics and appears to be more consistent across cohorts. It has been proved to be a more reliable and sensitive marker for estimating GFR in patients with normal creatinine-based eGFR (5) or with mildly renal insufficiency (6). According to the 2012 Kidney Disease: Improving Global Outcomes (KDIGO) guidelines (2), cystatin C-based eGFR may be used to verify the diagnosis of a reduced creatinine-based eGFR and, therefore, recategorize patients into normal group in the absence of albuminuria. Several studies have shown a very close relationship between cystatin C and cardiovascular events, especially cardiac death and all-cause mortality (6), among different cohorts, such as patients with chronic kidney disease(CKD) (7) and the elderly people (8). These studies have revealed that cystatin C-based eGFR can be an earlier and stronger predictor for predicting adverse cardiovascular outcomes as compared with creatinine-based eGFR.

Renal dysfunction can be estimated by various equations and has been identified as an important predictor of mortality for patients with coronary heart disease after percutaneous coronary intervention (PCI) and surgical revascularization. Chronic coronary total occlusion (CTO) is advanced atherosclerotic lesions and represents a challenging subset of coronary artery disease. However, the value of eGFR in predicting adverse cardiovascular events has not been well-established within the patients with CTO after PCI. This deficiency forms the basis for this study, aiming to determine whether or not the decreased eGFR calculated by cystatin C can have a higher risk predictive value for assessing risk and risk grading in terms of all-cause mortality and cardiac death in CTO cohort available in our Cardiology Department.

## Patients and Methods

### Population and Outcomes

A total of 762 patients with CTO lesions confirmed by coronary angiography in the Cardiology Department of the First Affiliated Hospital of Xi'an JiaoTong University (Xi'an, Shaanxi, China) undergoing PCI between June 2013 and October 2017 were included in the study. The follow-up for all-cause mortality and cardiac death was carried out via telephone contacts between 2018 and 2019. The study was approved by both Research and Ethics Committees of the First Affiliated Hospital of Xi'an JiaoTong University. Recanalization of CTO lesion was performed according to current guidelines (9, 10) by the highly experienced CTO operators with contemporary techniques. A coronary CTO lesion was defined as the presence of Thrombolysis in Myocardial Infarction [TIMI] flow grade 0 within an occluded coronary artery segment of an estimated duration of at least 3 months (11, 12). Procedural success was defined by complete restoration of antegraded blood flow (TIMI flow grade 3) in the occluded segment with <30% residual diameter stenosis. The primary endpoint was all-cause mortality, and the secondary endpoint was cardiac death.

### Measurements of Related Parameters

We collected several demographic, clinical, and analytical parameters. Age, gender, height, and weight were recorded, and body mass index (BMI) (kg/m^2^) was calculated on the basis of weight and height. Blood pressure (BP) was measured three times or more by a nurse while patients were seated after 5 min of rest. Patients either with persistent BP>140/90 mmHg or those currently taking antihypertensive drugs were considered hypertensive. Presence of type 2 diabetes mellitus (T2DM) was determined by means of a medical history of diabetes, fasting glucose levels >126 mg/dL (7.0 mmol/L), and/or glycated hemoglobin (HbA1c)>6.5%. Several parameters, including low-density lipoprotein-cholesterol (LDL-c), HDL cholesterol (HDL-c), and C-reactive protein, were measured. Serum levels of cystatin C were determined by an automated particle-enhanced immunoturbidimetric method on a Siemens Dade Behring BN II Nephelometer. Renal function was assessed by means of serum levels of creatinine and cystatin C at baseline and calculated by MDRD for the Chinese equation (13) (eGFR-cMDRD) and chronic kidney epidemiology collaboration (CKD-EPI) equations (4), respectively (Supplementary Table 1). CKD-EPI equations are composed of eGFR from serum creatinine (eGFRcre), eGFR from serum cystatin C (eGFRcys), and eGFR from the equation incorporating both creatinine and cystatin C (eGFRcre-cys).

### Categorization of the Cohorts

The cohort was categorized according to its prognostic category, defined by an existing classification system—the Kidney Disease: Improving Global Outcomes (KDIGO) guidelines (14)—that is, the level of GFR (known as stages). Therefore, patients were stratified according to eGFR (group 1: eGFR ≥ 90 ml/min/1.73 m^2^, group 2: 60–89 ml/min/1.73 m^2^, group 3: <60 ml/min/1.73 m^2^) as shown in Supplementary Table 2.

### Statistical Analysis

Continuous variables are presented as the mean ± standard deviation if normally distributed or median (lower quartile, upper quartile) otherwise, and Shapiro–Wilk was used for normality test. Categorical variables are presented as numbers and percentages. Differences in the parameters among groups were analyzed using analysis of variance (ANOVA) for normally distributed variables, the Kruskal–Wallis test was used for non-normally distributed continuous variables, and the chi-square test was used for categorical variables. We assessed performance through the receiver operating characteristic (ROC) curve and the area under the ROC curve (AUC). Clinical event rates were compared with the Kaplan–Meier method using the log rank test for comparisons between groups. Cox proportional hazards regression models were used in univariate analyses and multivariate analyses to determine the prognostic value of different kidney stage levels estimated by each eGFR equation. Multivariate analyses adjusted for significant baseline variables and the factors closely related to the outcome of patients with cardiovascular disease, such as age, sex, smoking, BMI, diabetes mellitus (DM), hypertension (HT), and low-density lipoprotein cholesterol (LDL-c), left ventricular ejection fraction (LVEF), C-reactive protein (CRP), and procedural success. Net reclassification improvement (NRI) and integrated discrimination improvement (IDI) were performed to analyze the degree to which eGFR improved the predictive ability of the eGFRcys. Both NRI and IDI values were analyzed with their 95% CI. SPSS version 25.0.0 (IBM, USA) and R version 3.6.1 (https://www.r-project.org) were used for conducting statistical analyses, and a two-sided *P* < 0.05 was considered statistically significant. There is no adjustment for multiplicity.

## Results

**Figure 1:**
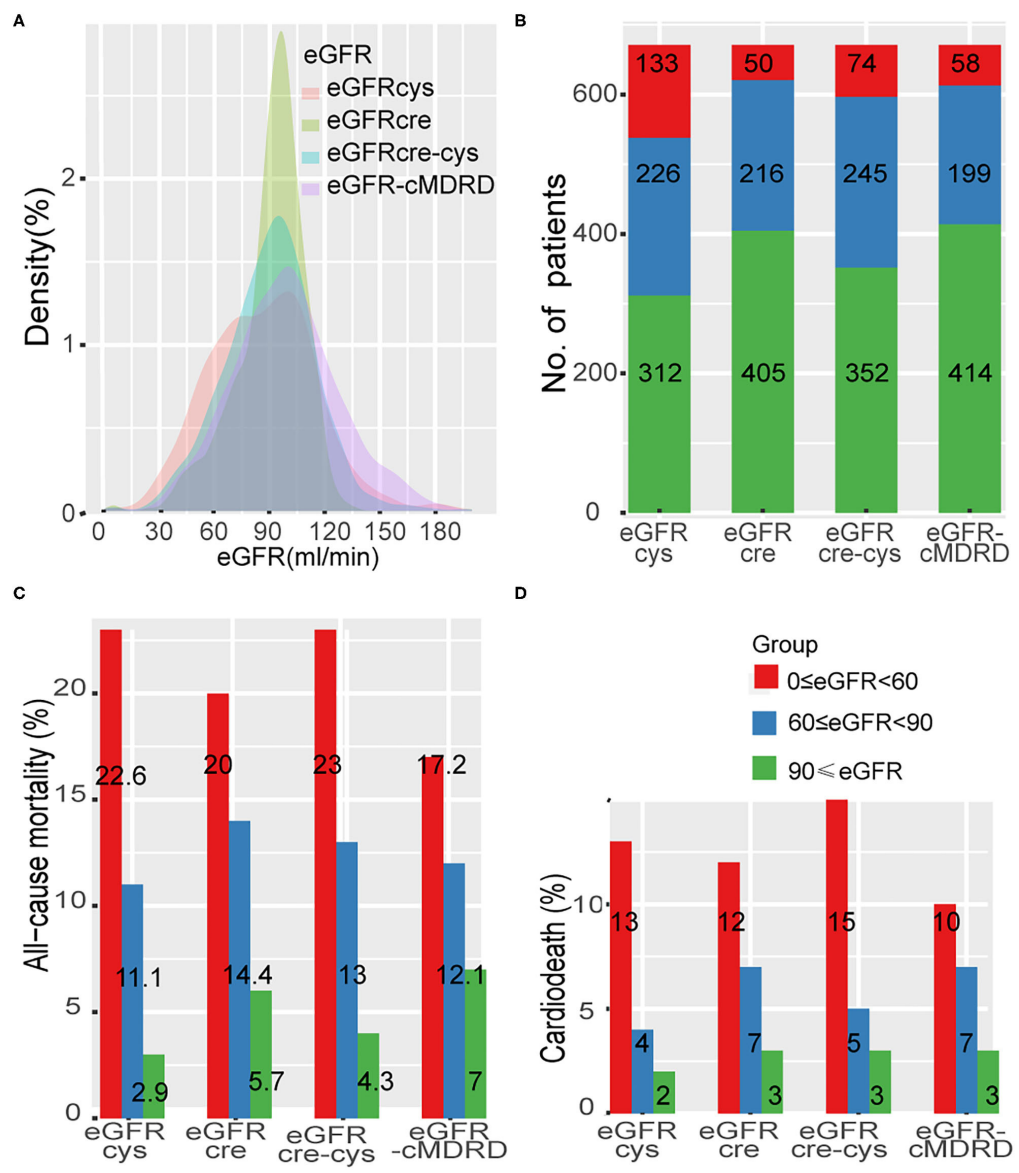
Distribution of eGFR and the rate of adverse event across different renal levels. **(A)**, Distribution of eGFR according to each of the four equations in a density plot. **(B)**, Thresholds of eGFR according to each equation and number of patients in each CKD stage. **(C,D)**, Incidence of all-cause mortality and cardiac death at different renal levels by different formulas. eGFR-cMDRD, calculated by MDRD for Chinese equation. eGFRcre, CKD-EPI equations composed of eGFR from serum creatinine; eGFRcys, eGFR from serum cystatin C; eGFRcre-cys, eGFR from equation incorporating both creatinine and cystatin C.

A total of 97 patients with acute coronary syndrome at admission were excluded. A total of 762 patients with CTO lesions confirmed by coronary angiography were treated with PCI, and the rate of CTO lesion revascularized was 70.2%. After a median follow-up of 33 months, 91 patients were lost to follow-up, leaving 671 in the study population at the end of follow-up (Supplementary Figure 1). In our cohort, 64 patients (9.5%) died and 33 patients (4.9%) died from cardiac death. Distribution of eGFR according to each of the four equations is shown in Figure 1A. Differences in grouping situation of four equations are shown in Figure 1B.

The baseline characteristics of the cohort are shown in Table 1. With decrease of renal function, patients were older (*p* < 0.001) with fewer men (*p* < 0.001) and had a higher prevalence of smoking (*p* < 0.01), a lower (although within normal range) ejection fraction (*p* < 0.001), and increased levels of CRP as well. Procedural success rate, prevalence of DM and HT, serum levels of LDLc, and other baseline parameters did not differ among the three groups.

**Table 1:** Baseline characteristics, by GFR, estimated using the eGFRcys equation.

	**eGFRcys > 90 (ml/min/1.73 m2)**	**eGFRcys 90–60 (ml/min/1.73 m2)**	**eGFRcys <60 (ml/min/1.73 m2)**	***p***
Patients	312 (46.5%)	226 (33.7%)	133 (19.8%)	
Age (years)	62 (54–68)	68 (61–73)	72 (66–78)	<0.001
Sex (men)	271 (86.9%)	195 (86.3%)	95 (71.4%)	<0.001
BMI	24.2 (22.8–26.2)	24.2 (22.6–25.7)	24.2 (23.4–25.4)	0.12
Drunk	77 (24.7%)	50 (22.1%)	22 (16.5%)	0.17
Smoking	166 (53.2%)	116 (51.3%)	50 (37.6%)	0.008
Previous MI	96 (30.8%)	76 (33.6%)	45 (33.8%)	0.77
Procedural success	220 (70.5%)	157 (69.5%)	94 (70.7%)	0.96
Multivessel disease				0.14
Single vessel	30 (9.60%)	15 (6.60%)	5 (3.80%)	
Double vessel	60 (19.2%)	35 (15.5%)	22 (16.5%)	
Triple vessel	222 (71.2%)	176 (77.9%)	106 (79.7%)	
J-CTO score				0.097
0	21 (6.7%)	7 (3.1%)	4 (3.0%)	
1	42 (13.5%)	22 (9.7%)	17 (12.8%)	
2	84 (26.9%)	54 (23.9%)	28 (21.1%)	
≥3	165 (52.9%)	143 (63.3%)	84 (63.2%)	
Stroke	25 (8.00%)	30 (13.3%)	13 (9.80%)	0.14
DM	144 (36.5%)	72 (31.9%)	52 (39.1%)	0.33
HT	179 (57.4%)	112 (49.6%)	82 (61.7%)	0.06
HR	70 (65–74)	70 (66–75)	70 (67–76)	0.14
SysBP (mmHg)	125 (118–135)	124 (116–134)	127 (117–140)	0.19
DiaBP (mmHg)	73 (67.1–78.8)	72 (66–77.6)	72 (66–78.3)	0.59
CRP (mg/L)	1.40 (0.800–1.40)	1.40 (1.28–1.80)	1.40 (1.40–3.20)	<0.001
WBC (10^9^/L)	6.39 (5.42–7.81)	6.22 (5.33–7.01)	6.41 (5.32–7.56)	0.15
Neutrophils (10^9^/L)	4.00 (3.33–5.07)	4.03 (3.21–4.95)	4.23 (3.40–5.17)	0.25
Cholesterol (mmol/L)	3.71 (3.08–4.33)	3.61 (3.05–4.17)	3.64 (3.07–4.58)	0.38
Triglyceride (mmol/L)	1.47 (1.02–2.10)	1.40 (0.980–1.96)	1.38 (1.04–1.95)	0.53
HDL (mmol/L)	0.910 (0.790–1.08)	0.920 (0.800–1.04)	0.950 (0.800–1.10)	0.57
LDLc (mmol/L)	2.12 (1.66–2.66)	2.01 (1.56–2.58)	2.09 (1.56–2.79)	0.40
LVEF (%)	60.0 (48.3–80.0)	58.5 (46.0–47.0)	53.0 (42.0–63.0)	<0.001

Values are given as median and interquartile range or numbers and percentages.

*BMI, body mass index; LVEF, left ventricular ejection fraction; DM, diabetes mellitus; HT, hypertension; CRP, C-reactive protein; HR, heart rate; SysBP, systolic blood pressure; DiaBP, diastolic blood pressure; eGFR, estimated glomerular filtration rate; eGFRcys, eGFR from serum cystatin C*.

Levels of renal function estimated by each eGFR equation (eGFR-cMDRD, eGFRcys, eGFRcre, and eGFRcre-cys) were classified according to the risk event of all-cause mortality and cardiac death (Figures 1C,D, Supplementary Table 3). All the groups showed a trend that all-cause mortality was increased with the decreased renal function (eGFRcys: group 1 to group 3, 2.9–22.6%; eGFRcre: group 1 to group 3 5.7–20.0%; eGFRcre-cys: group 1 to group 3, 4.3–23.0%; and eGFR-MDRD: group 1 to group 3, 7.0–17.2%). The same was true when cardiac death rates were tested (eGFRcys: group 1 to group 3, 1.90–12.80%; eGFRcre: group 1 to group 3, 2.70–12.00%; eGFRcre-cys: group 1 to group 3, 2.60–14.90%; and eGFR-MDRD: group 1 to group 3, 3.4–10.3%).

**Figure 2:**
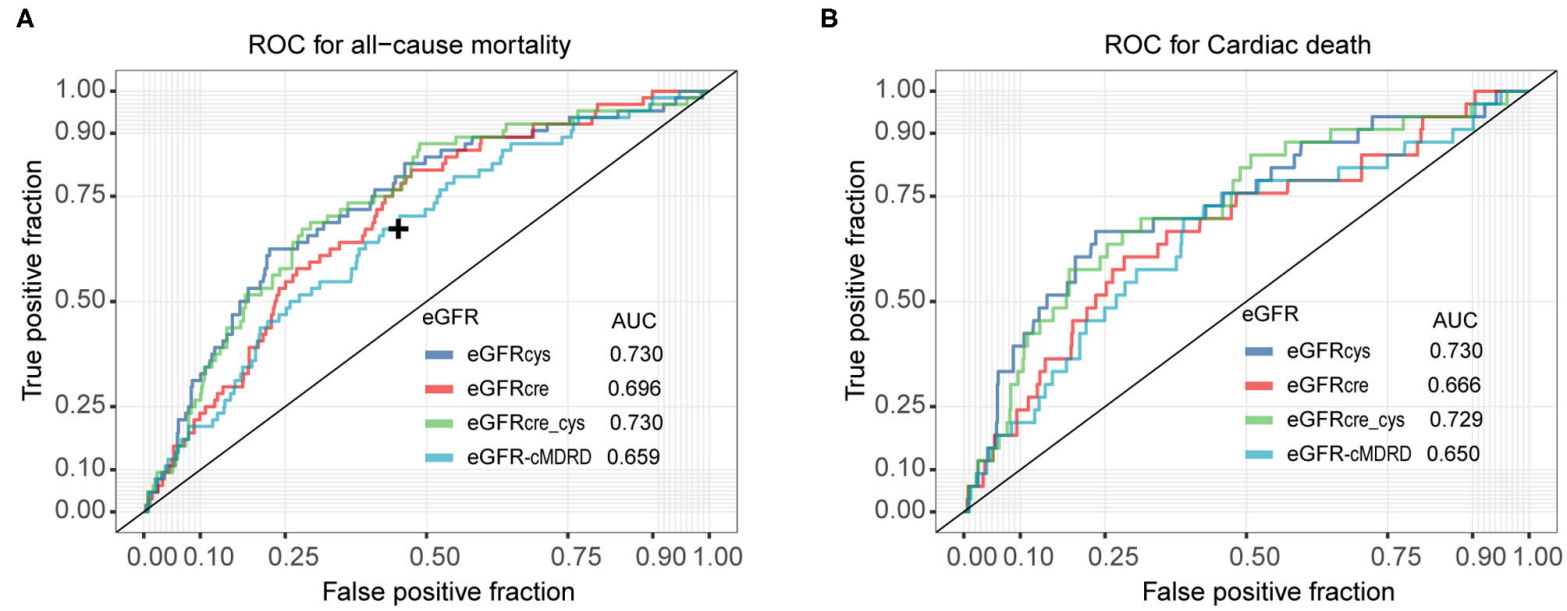
ROC for predicting all-cause mortality and cardiac death. **(A)**, accuracies of eGFRcys, eGFRcre, eGFRcre-cys, and eGFR-cMRDR for predicting all-cause mortality presented as areas under the receiver operating characteristic curves, individually. **(B)**, accuracies of eGFRcys, eGFRcre, eGFRcre-cys, and eGFR-cMRDR for predicting cardiac death presented as areas under the receiver operating characteristic curves, separately. ROC, receiver operating characteristic curve; AUC, area under the receiver operating characteristic curve. DeLong's test to check the difference of AUC for eGFRs calculated by other formulas, compared to eGFRcys. + represents significant differences (*P* < 0.05).

The accuracies of eGFR-cMDRD, eGFRcys, eGFRcre, and eGFRcre-cys for predicting all-cause mortality and cardiac death are shown as the AUC in Figure 2, Supplementary Table 4. For predicting all-cause mortality, the eGFRcys (AUC = 0.73) and eGFRcre-cys (AUC = 0.73) exhibited higher predictive accuracy (Figure 2A). For predicting cardiac death, eGFRcys (AUC = 0.73) showed the best prediction performance (Figure 2B).

**Figure 3:**
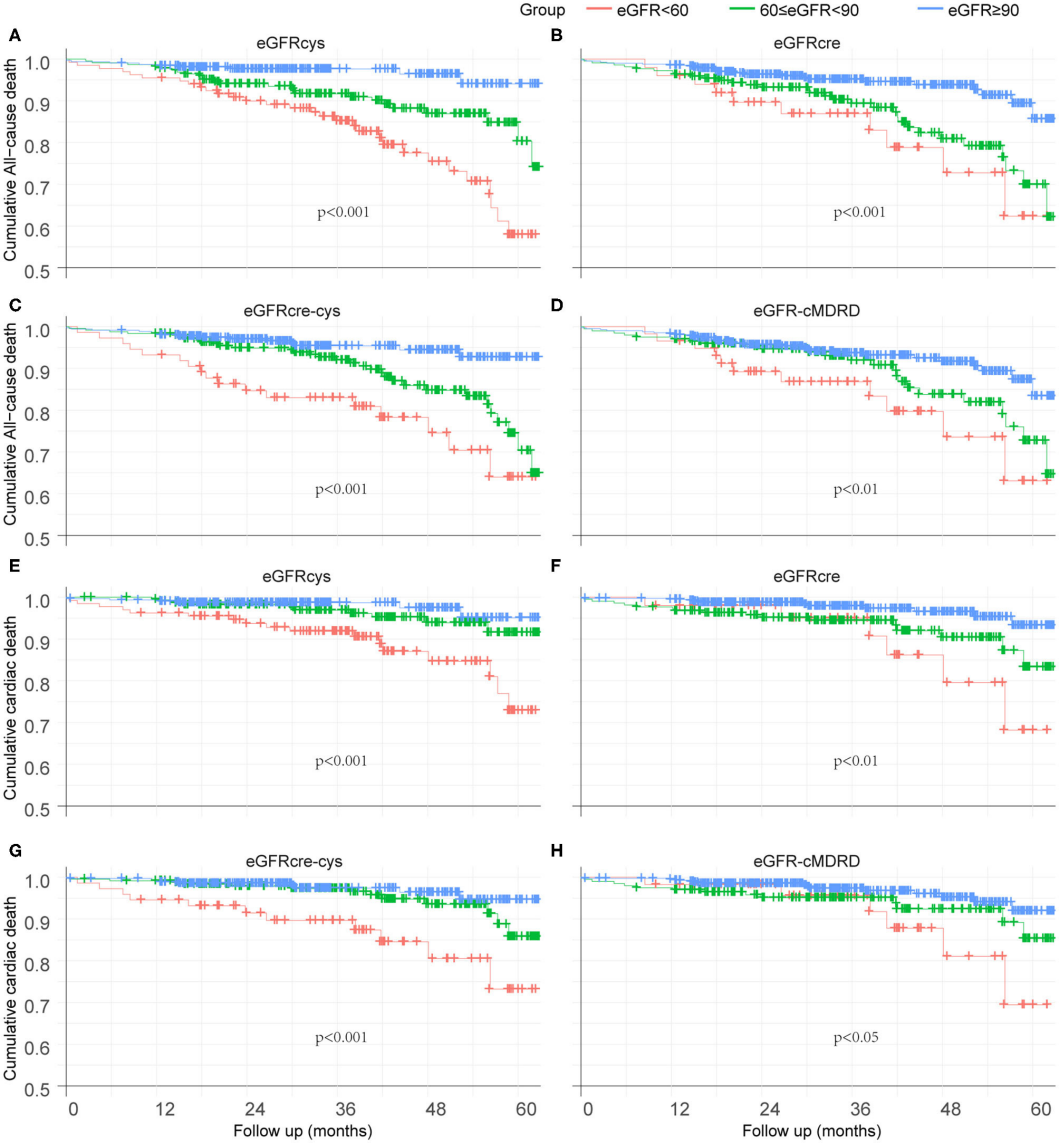
Kaplan–Meier curves to illustrate the survival free from adverse event. **(A–D)** Survival analysis of eGFRcys, eGFRcre, eGFRcre-cys, and eGFR-cMRDR for all-cause mortality, individually. **(E–H)** Survival analysis of eGFRcys, eGFRcre, eGFRcre-cys, and eGFR-cMRDR for cardiac death, separately. No. at risk = number of patients at risk for all-cause mortality or cardiac death.

Kaplan–Meier curves were used to illustrate the survival free from adverse events. Overall, patients at a lower eGFR levels had a significantly worse outcome of survival free from all-cause mortality during the follow-up period (Figures 3A–D). eGFRcys showed better performance on grading and risk assessment. Alike, the lower renal function is, the worse survival free from cardiac death is (Figures 3E–H). Also, eGFRcys showed the superior performance on grading and risk assessment as well as eGFRcre-cys to other equations.

We performed Cox regression hazard models on eGFR calculated by each equation as the categorical variable grouped as previously mentioned. The risk of each adverse outcome was assessed by comparing Group 1 (reference) with Groups 2 and 3 in every equation. Cystatin C-based eGFR (adjusted HR, 3.6; 95% CI, 1.6–8.1; *P* = 0.002) was a stronger predictor than eGFRcre (adjusted HR 1.7, 95% CI 0.7–4.1, *P* = 0.203), eGFRcre-cys (adjusted HR, 2.3; 95% CI, 1.0–5.3; *P* = 0.049), and eGFR-cMDRD (adjusted HR, 1.3; 95% CI, 0.6–2.9; *P* = 0.516) for the risk of all-cause mortality for moderate-to-severe reduction in GFR (eGFR <60 ml/min/1.73 m^2^). Likewise, cystatin C-based eGFR (adjusted HR, 2.9; 95% CI, 1.0–8.1; *P* = 0.028) was able to predict the risk of cardiovascular mortality better than eGFRcre (adjusted HR, 2.3; 95% CI, 0.7–7.6; *P* = 0.155), eGFRcre-cys (adjusted HR, 2.4; 95% CI, 0.8–7.2; *P* = 0.12), and eGFR-cMDRD (adjusted HR, 1.5; 95% CI, 0.5–4.2; *P* = 0.498) for moderate-to-severe reduction in renal function.

**Figure 4:**
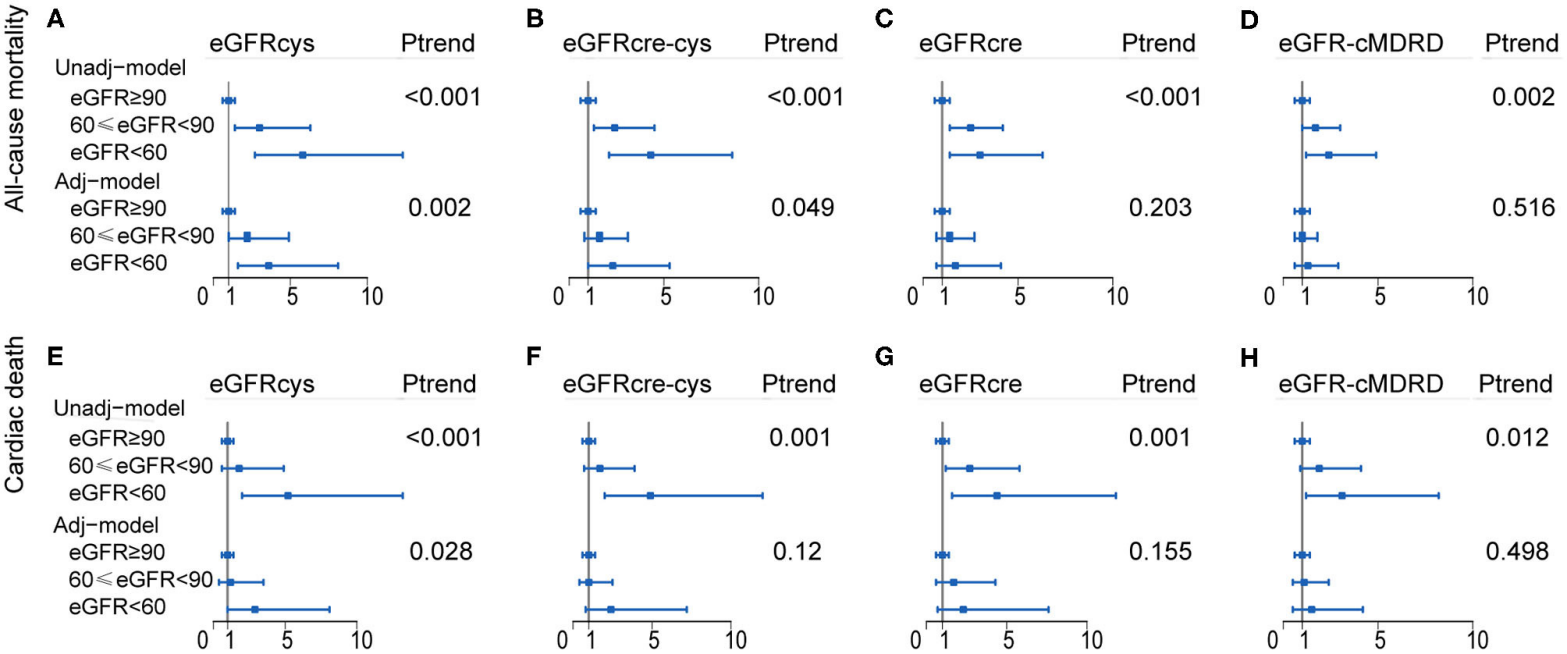
Forest plot showing the risk assessment of event across renal levels. HR and 95% CI of the association between both all-cause mortality **(A–D)** and cardiac death **(E–H)** and the eGFR calculated by of four equations. Group 1 (eGFR ≥ 90 ml/min/1.73 m2) as reference group, Group 2 (60 ≤ eGFR <90 ml/min/1.73 m2), and Group 3 (eGFR <60 ml/min/1.73 m2) were compared with it, separately. Adj-model was adjusted for baseline variables significantly and factors closely related to the outcome of patients with cardiovascular disease, such as age, sex, smoking, body mass index (BMI), diabetes mellitus (DM), hypertension (HT), and low-density lipoprotein cholesterol (LDL-c), left ventricular ejection fraction (LVEF), C-reactive protein (CRP), and procedural success. Unadj-model, Unadjusted model; Adj-model, Adjusted model; Ptrend, P for trend.

The above data showed that in terms of outcome of all-cause mortality, eGFRcre was able to estimate an increased risk that was 1.7 times higher than that of the population without CKD (patients whose eGFR was above 90 ml/min/1.73 m^2^). On the other hand, eGFRcys showed an increased risk rate of 3.6-folds, respectively, as compared with that of the population without CKD. This means that cystatin C is able to improve the predictive value by 111.7% (for the eGFRcys) in patients with moderate-to-severe reduction in GFR in terms of all-cause mortality. Likewise, cystatin C-based eGFR may improve the estimation of risk by 26.1% (for the cystatin C-based equation) in terms of cardiovascular mortality, as compared with those estimated with other equations. A forest plot summarizing these data is shown in Figure 4, Supplementary Tables 5, 6.

**Figure 5:**
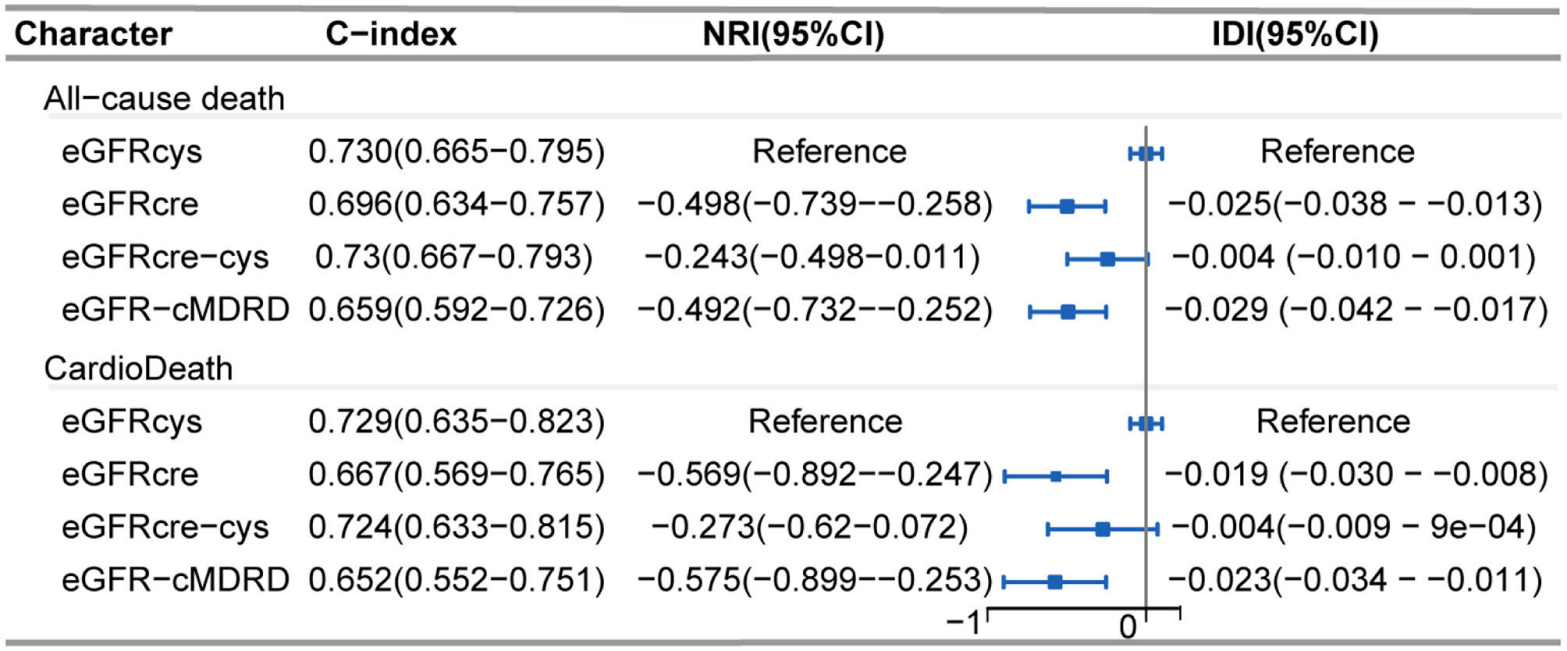
Forest plot showing net reclassification improvement of renal function calculated by various eGFR equations. Net reclassification improvement in all-cause mortality and cardiac death was assessed by comparing eGFRcre, eGFRcre-cys, and eGFR-cMRDR with eGFRcys (reference), individually. C-index: Harrell's concordance index. NRI, Net reclassification improvement; IDI, integrated discrimination improvement; CI, confidence interval.

Regarding discrimination, NRI and IDI in all-cause mortality and cardiac death were assessed by comparing eGFRcre, eGFRcre-cys, and eGFR-cMDRD to eGFRcys (reference). Overall, the reclassification of other equations performed worse both on all-cause mortality and cardiac death (Figure 5). Cystatin C-based eGFR also showed the best performance with the maximum C-index (Figure 5) both in all-cause mortality and cardiac death.

## Discussion

This study showed an increase in all-cause mortality and cardiac death after CTO PCI as baseline renal function declined. Patients with renal insufficiency represent a high-risk patient subset. The values of the four measures showed similar accuracy using the area under the receiver operating characteristic curves. The classification into standard kidney stages showed significant differences in the survival curves and Cox regression models for both endpoints.

Given the rapidly aging population in the majority of regions of the world, along with the growing prevalence of chronic disease, chronic kidney disease has become a significant contributor to the increased morbidity and mortality (1). Consistent with the findings made in previous studies for patients suffering from both cardiovascular disease and renal impairment (15, 16), we found that patients age and the prevalence of adverse cardiovascular event were increased across stages of renal dysfunction in patients received CTO-PCI. Patients with renal impairment more frequently presented with a significantly lower (although within normal range) ejection fraction (*p* < 0.001) and increased levels of CRP. We also found some HR *p*-values which are significant in the univariate analyses become non-significant after adjusting for confounders, and this statistical phenomenon mostly occurred in creatinine-based eGFR. The true causal effect between eGFR values and outcome is affected by confounders (17), defined as variables that are associated with both eGFR values and outcome, but influenced by neither. Differences among the eGFR values with respect to risk relationships probably reflect confounding by non-GFR determinants of the filtration markers. It is well-known that the non-GFR determinants of serum creatinine, including age, sex, BMI, DM, and others can confound the associations between the creatinine-based eGFR and outcomes (18). Non-GFR determinants of cystatin C also exist, though they are quantifiably smaller than those of creatinine (19).

The main novelty of our research centers on the fact that it was specifically performed in patients with CTO. To the best of our knowledge, this is the first study to address the prognostic usefulness of eGFRcys, by comparing it with eGFRcrea, eGFRcre-cys, and eGFR-cMDRD in patients that received CTO PCI. The analysis of our series of patients suggested that eGFRcys might be a better predictor for cardiovascular events in patients with renal impairment (<60 ml/min/1.73 m^2^) when compared with the individuals with normal renal function (>90 ml/min/1.73 m^2^). The main finding in our research is that the eGFRcys performed better than other three eGFR equations in assessing the adverse cardiovascular risk in both cardiac death and all-cause mortality in our CTO population.

Consistent with our findings, Shlipak et al. (7, 20) found that serum levels of cystatin C were strongly predictive of all-cause mortality and cardiovascular disease risk, whereas serum levels of creatinine showed relatively poorer performance in predicting adverse outcomes. Peralta et al. (21) demonstrated that the eGFRcys played a more important role than eGFRcre in identifying the high risk of adverse outcome, both in patients with a moderate to severe renal insufficiency (eGFR <60 ml/min/1.73 m^2^) and in patients with a mild renal insufficiency (60–89 ml/min/1.73 m^2^). Recently, Shlipak et al. (6) demonstrated the increased risk of death for eGFRcys for patients with a mild reduction in renal function (60–89 ml/min/1.73 m^2^). Pedro et al. (22) provided the evidence suggesting that the use of eGFRcys improved the role of eGFR in risk stratification of all-cause mortality for patients with non-ST-segment elevated acute coronary syndrome, as compared with eGFR-MDRD. Helmersson-Karlqvist et al. (23) and Lim et al. (24) found that cystatin C-based eGFR was more closely associated with mortality as compared with both eGFRcre and eGFRcre-cys. Peralta et al. (21) also found the benefits of the eGFRcys in reclassifying mortality risk among adults diagnosed with CKD using the eGFRcre who had the highest risk for complications. Similar clinical applications of cystatin C in a subset of patients would be its use as a confirmatory test, where cystatin C would predict risk for all-cause mortality and cardiovascular events among hypertensive patients (25). Thus, above results are consistent with our findings that eGFRcys can improve the risk-grading by identify patients at different levels of risk for adverse outcome whose risk has been estimated with eGFRcre.

## Strengths

One strength of our research was the use of eGFRcys to estimate the risk of cardiac death and all-cause mortality with standard statistical methods, which could be better than the use of the other equations. It should be noted that of more significance to our study was the assessment of a distinct subset of population, i.e., CTO patients. Our results may provide physicians with a support for the need of reconsidering which eGFR equation is the best one for assessment of long-term adverse events in CTO patients.

## Limitations

The research is a single-center retrospective observational design and is restricted in generalization of the findings. Future multicenter prospective study with a larger number of patients and a longer follow-up is required.

## Conclusion

This study has demonstrated that all-cause mortality and cardiac death are increased across stages of renal dysfunction, and the decreased eGFR calculated with cystatin C has a higher risk predictive value to assessing risk and risk-grading in terms of all-cause mortality and cardiac death in our CTO population than eGFR calculated by other three equations.

## Data Availability Statement

The data generated during this study are included in this article. Raw data are available upon reasonable request.

## Ethics Statement

The studies involving human participants were reviewed and approved by Research and Ethics Committees of the First Affiliated Hospital of Xi'an JiaoTong University. The patients/participants provided their written informed consent to participate in this study.

## Author Contributions

BL: conceptualization, project administration, writing - original draft, and writing - review and editing. JR: methodology, project administration, data analysis, and writing - review and editing. BW, KG, Y-MH, SL, and ZJ: investigation and data curation. XW: data curation and writing - review. HL: resources and data curation. LC: writing - review and editing. YZ and HH: writing - review and editing and data curation. YP: data curation. YW: resources, funding acquisition, and writing - review and editing. X-ZZ: supervision, project administration, and writing - review and editing. All authors contributed to the article and approved the submitted version.

## Conflict of Interest

The authors declare that the research was conducted in the absence of any commercial or financial relationships that could be construed as a potential conflict of interest.
