# Supplementary material for: Cystatin C-Based Renal Function in Predicting the Long-Term Outcomes of Chronic Total Occlusion After Percutaneous Coronary Intervention
Source: Front Cardiovasc Med. 2020 Nov 9;7:586181. doi: 10.3389/fcvm.2020.586181 (PMC7693449; doi:10.3389/fcvm.2020.586181)
Supplement: Supplementary file 1 [file Table_1.DOCX]

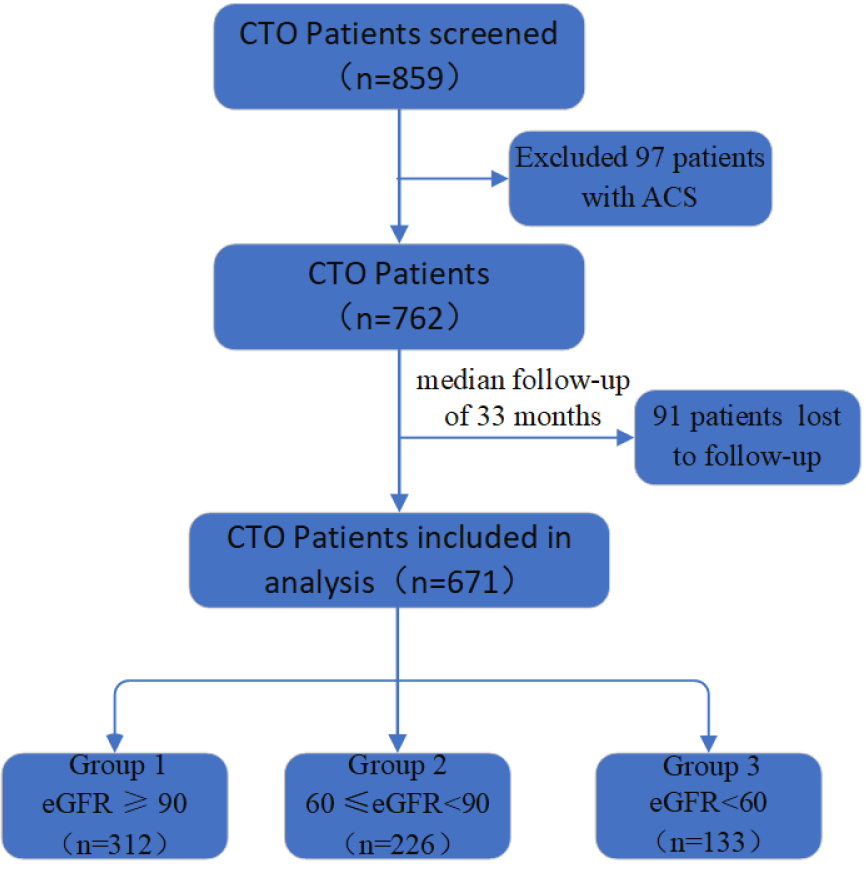


**Supplementary figure 1** Study Design. CTO = Chronic coronary total occlusion. ACS = acute coronary syndrome. eGFR = estimated glomerular ﬁltration rate.


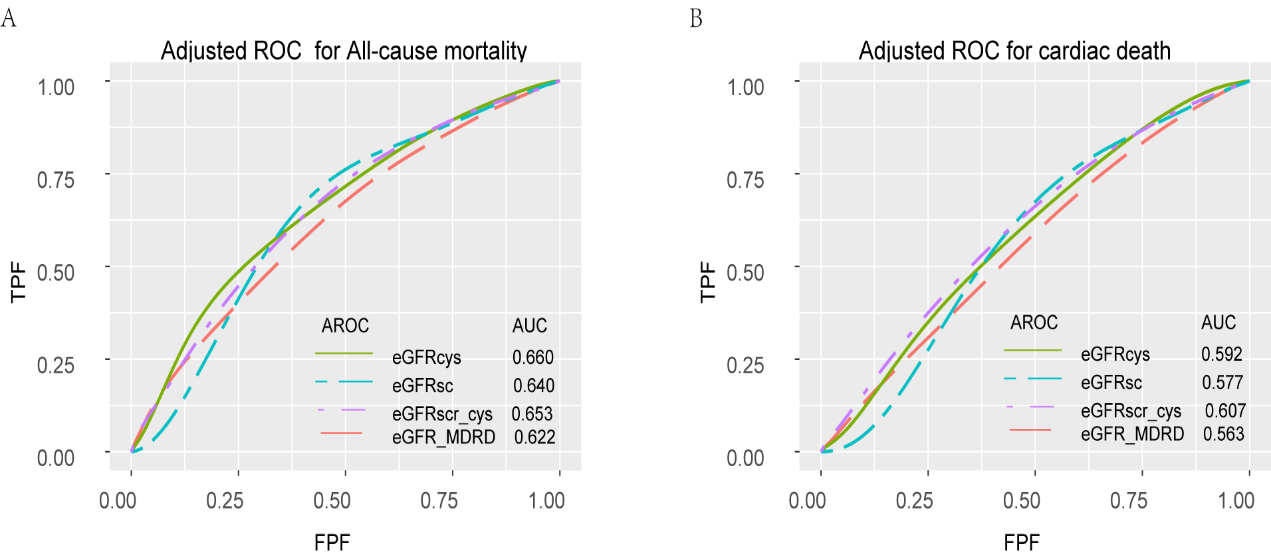


**Supplementary figure 2** Adjusted ROC for predicting all-cause mortality and cardiac death. A, accuracies of eGFRcys, eGFRcre, eGFRcre-cys, eGFR-cMRDR adjusted age, sex, LVEF and CRP for predicting all-cause mortality presented as areas under the receiver operating characteristic curves, individually. B, accuracies of eGFRcys, eGFRcre, eGFRcre-cys, eGFR-cMRDR adjusted age, sex, LVEF and CRP for predicting cardiac death presented as areas under the receiver operating characteristic curves, separately. ROC= receiver operating characteristic curve. AUC=area under the receiver operating characteristic curve.

| **Supplementary table 1** Different eGFR equations. | | | | |
| --- | --- | --- | --- | --- |
| eGFR | Sex | Crea (mg/dL) | Cys-C (mg/L) | Equation (ml/min/1.73m^2^) |
| CKD-EPI_cre_  (eGFR_cre_) | Female | ≤0.7 |  | 144×(Cre / 0.7) ^- 0.329^ × 0.993 ^Age^ × 1.018 |
|  |  | >0.7 |  | 144 × (Cre / 0.7) ^- 1.209^ × 0.993^Age^ × 1.018 |
|  | Male | ≤0.9 |  | 141 × (Cre / 0.9) ^- 0.411^ × 0.993^Age^ |
|  |  | >0.9 |  | 141 × (Cre / 0.9) ^- 1.209^ × 0.993^Age^ |
| CKD-EPI_cys_  (eGFR_cys_) | Female |  | ≤0.8 | 133 × (Cys / 0.8) ^- 0.499^ × 0.996^Age^ × 0.932 |
|  |  |  | >0.8 | 133 × (Cys / 0.8) ^- 1.328^ × 0.996^Age^ × 0.932 |
|  | Male |  | ≤0.8 | 133 × (Cys / 0.8) ^- 0.499^ × 0.996^Age^ |
|  |  |  | >0.8 | 133 × (Cys / 0.8) ^- 1.328^ × 0.996^Age^ |
| CKD-EPI_cre-cys_  (eGFR_cre-cys_) | Female | ≤0.7 | ≤0.8 | 130 × (Cre / 0.7) ^- 0.248^ (Cys / 0.8) ^- 0.375^ ×0.995^Age^ |
|  |  |  | >0.8 | 130 × (Cre / 0.7) ^- 0.248^ × (Cys / 0.8) ^- 0.711^ ×0.995^Age^ |
|  |  | >0.7 | ≤0.8 | 130 × (Cre / 0.7) ^- 0.601^ × (Cys / 0.8) ^- 0.375^ ×0.995^Age^ |
|  |  |  | >0.8 | 130 × (Cre / 0.7) ^- 0.601^ × (Cys / 0.8) ^- 0.711^ ×0.995^Age^ |
|  | Male | ≤0.9 | ≤0.8 | 135 × (Cre / 0.9) ^- 0.207^ ×(Cys / 0.8) ^- 0.375^ ×0.995^Age^ |
|  |  |  | >0.8 | 135 × (Cre / 0.9 ) ^- 0.207^ ×(Cys / 0.8) ^- 0.711^ ×0.995^Age^ |
|  |  | >0.9 | ≤0.8 | 135 ×(Cre / 0.9) ^- 0.601^ ×(Cys / 0.8) ^- 0.375^ ×0.995^Age^ |
|  |  |  | >0.8 | 135 ×(Cre / 0.9) ^- 0.601^ ×(Cys / 0.8) ^- 0.711^ ×0.995^Age^ |
| MDRD for Chinese Patients  (eGFR-cMDRD) | Female |  |  | 175 ×(Cre / 0.9) ^- 1.234^ × (Age) ^- 0.179^ ×0.79 |
|  | Male |  |  | 175 ×(Cre / 0.9) ^- 1.234^ × (Age) ^- 0.179^ |

The eGFR calculated by modification of diet in renal disease equation for Chinese (cMDRD) and Chronic Kidney Disease Epidemiology Collaboration (CKD-EPI) equations. MDRD for Chinese equation (eGFR-cMDRD). CKD-EPI equations composed of eGFR from serum creatinine (eGFRcre), eGFR from serum cystatin C (eGFRcys) and eGFR from equation incorporating both creatinine and cystatin C (eGFRcre-cys).

| **Supplementary table 2** Thresholds of eGFR according to each equation and number of patients in each group clinical CKD stages | | | | |
| --- | --- | --- | --- | --- |
| Clinical CKD stages (ml/min/1.73m^2^) | GFR equations | | | |
|  | eGFRcys | eGFRcre | eGFRcre-cys | eGFR-MDRD |
| Group1:≥90 | 312(46.5%) | 405(60.4%) | 352(52.2%) | 414(61.7%) |
| Group2:90-60 | 226(33.7%) | 216(32.2%) | 245(36.5%) | 199(29.7%) |
| Group3:≤60 | 133(19.8%) | 50(7.5%) | 74(11.0%) | 58(8.6%) |

| **Supplementary table 3** Incidence of all-cause mortality and cardiac Death | | | | |
| --- | --- | --- | --- | --- |
|  | eGFR ≥ 90  (ml/min/1.73m2) | eGFR 90-60  (ml/min/1.73m2) | eGFR < 60  (ml/min/1.73m2) | *p* for trend |
| All-cause mortality |  |  |  |  |
| eGFRcys | 9(2.90%) | 25(11.1%) | 30(22.6%) | <0.001 |
| eGFRcre | 23(5.70%) | 31(14.4%) | 10(20.0%) | <0.001 |
| eGFRcre-cys | 15(4.30%) | 32(13.1%) | 17(23.0%) | <0.001 |
| eGFR-cMDRD | 29(7.00%) | 24(12.1%) | 11(17.2%) | 0.005 |
| Cardiac Death |  |  |  |  |
| eGFRcys | 6(1.9%) | 10(4.4%) | 17(12.8%) | <0.001 |
| eGFRcre | 11(2.7%) | 16(7.4%) | 6(12.0%) | 0.002 |
| eGFRcre-cys | 9(2.6%) | 13(5.3%) | 11(14.9%) | <0.001 |
| eGFR-cMDRD | 14(3.4%) | 13(6.5%) | 6(10.3%) | 0.033 |

**Supplementary table 4** De Long's test for AUC

|  | **AUC** | *p*  (De Long's test) | |
| --- | --- | --- | --- |
| **ROC for All-cause mortality** |  |  | |
| eGFRcys | 0.730 | reference | |
| eGFRcre | 0.696 | 0.236 | |
| eGFRcre-cys | 0.730 | 0.979 | |
| eGFR-cMDRD | 0.659 | 0.024 | |
| **ROC for cardiac death** | | |  |
| eGFRcys | 0.730 | reference | |
| eGFRcre | 0.666 | 0.148 | |
| eGFRcre-cys | 0.729 | 0.687 | |
| eGFR-cMDRD | 0.650 | 0.081 | |

DeLong's test to check the difference of AUC for eGFRs calculated by other formulas, compared to eGFRcys. AUC=area under the receiver operating characteristic curve.

| **eGFR** | HR (95%CI) | | | |
| --- | --- | --- | --- | --- |
|  | Group1 | Group2 | Group3 | *p* for trend |
| **eGFRcys** |  |  |  |  |
| Unadj-model | 1 | 3.0(1.4-6.3) | 5.8(2.7-12.3) | <0.001 |
| Adj-model | 1 | 2.2(1.0-4.9) | 3.6(1.6-8.1) | 0.002 |
| **eGFRcre** |  |  |  |  |
| Unadj-model | 1 | 2.5(1.4-4.2) | 3.0(1.4-6.3) | <0.001 |
| Adj-model | 1 | 1.4(0.7-2.7) | 1.7(0.7-4.1) | 0.203 |
| **eGFRcre-cys** |  |  |  |  |
| Unadj-model | 1 | 2.4(1.3-4.5) | 4.3(2.1-8.6) | <0.001 |
| Adj-model | 1 | 1.6(0.8-3.1) | 2.3(1.0-5.3) | 0.049 |
| **eGFR-cMDRD** |  |  |  |  |
| Unadj-model | 1 | 1.7(1.0-3.0) | 2.4(1.2-4.9) | 0.002 |
| Adj-model | 1 | 1.0(0.6-1.8) | 1.3(0.6-2.9) | 0.516 |

**Supplementary table 5** The risk assessment of all-cause mortality across renal levels

HR and 95% CI of the association between all-cause mortality and the eGFR calculated by of 4 equations. Group 1 (eGFR≥90 ml/min/1.73 m2) as reference group, Group 2 (60≤eGFR<90 ml/min/1.73 m2) and Group 3 (eGFR＜60 ml/min/1.73 m2) were compared with it, separately. Adj-model was adjusted for baseline variables significantly and factors closely related to the outcome of patients with cardiovascular disease, such as age, sex, smoking, body mass index (BMI), diabetes mellitus (DM), hypertension (HT) and low density lipoprotein cholesterol (LDL-c), Left ventricular ejection fraction (LVEF), C-reactive protein (CRP) and procedural success. Unadj-model = Unadjusted model. Adj-model = Adjusted model.

| **eGFR** | HR (95%CI) | | | |
| --- | --- | --- | --- | --- |
|  | Group1 | Group2 | Group3 | *p* for trend |
| **eGFRcys** |  |  |  |  |
| Unadj-model | 1 | 1.8(0.6-4.9) | 5.2(2.0-13.2) | <0.001 |
| Adj-model | 1 | 1.2(0.4-3.5) | 2.9(1.0-8.1) | 0.028 |
| **eGFRcre** |  |  |  |  |
| Unadj-model | 1 | 2.7(1.2-5.8) | 4.4(1.6-11.8) | 0.001 |
| Adj-model | 1 | 1.7(0.6-4.3) | 2.3(0.7-7.6) | 0.155 |
| **eGFRcre-cys** |  |  |  |  |
| Unadj-model | 1 | 1.7(0.7-3.9) | 5.0(2.0-12.0) | 0.001 |
| Adj-model | 1 | 1.0(0.4-2.5) | 2.4(0.8-7.2) | 0.12 |
| **eGFR-cMDRD** |  |  |  |  |
| Unadj-model | 1 | 1.9(0.9-4.1) | 3.1(1.2-8.2) | 0.012 |
| Adj-model | 1 | 1.1(0.5-2.4) | 1.5(0.5-4.2) | 0.498 |

**Supplementary table 6** The risk assessment of cardiac death across renal levels

HR and 95% CI of the association between cardiac death and the eGFR calculated by of 4 equations. Group 1 (eGFR≥90 ml/min/1.73 m2) as reference group, Group 2 (60≤eGFR<90 ml/min/1.73 m2) and Group 3 (eGFR＜60 ml/min/1.73 m2) were compared with it, separately. Adj-model was adjusted for baseline variables significantly and factors closely related to the outcome of patients with cardiovascular disease, such as age, sex, smoking, body mass index (BMI), diabetes mellitus (DM), hypertension (HT) and low density lipoprotein cholesterol (LDL-c), Left ventricular ejection fraction (LVEF), C-reactive protein (CRP) and procedural success. Unadj-model = Unadjusted model. Adj-model = Adjusted model.
